# High salinity selects for halotolerant gut microbiota in the mummichog *Fundulus heteroclitus*

**DOI:** 10.17912/micropub.biology.002267

**Published:** 2026-10-12

**Authors:** Reece Bradberry, Elyse Hutchinson, Carlie Edwards, James Wetzel, Austin Y Shull, Stuart Gordon

**Affiliations:** 1 Department of Biology, Presbyterian College, Clinton, SC, United States

## Abstract

Mummichogs ( *Fundulus heteroclitus* ) are euryhaline fish that maintain osmoregulation across a range of salinities, but how their gut microbiomes respond remains poorly understood. Using Illumina 16S amplicon and Oxford Nanopore full-length 16S sequencing, we show that salinity is associated with reproducible gut microbiome restructuring. While alpha diversity did not differ significantly between low-salinity (5 ppt) and high-salinity (50 ppt) groups, beta diversity revealed distinct shifts in microbial communities. Specifically, high-salinity microbiomes were dominated by halotolerant *Vibrio* and *Ahrensia* species while low-salinity microbiomes retained more taxonomically diverse communities. These findings suggest host-associated microbes may contribute to osmotic homeostasis under salinity stress.

**Figure 1. Environmental salinity shapes gut microbiome composition in mummichogs ( Fundulus heteroclitus ):** **A)** Experimental design illustrating mummichogs maintained at low (5 ppt) or high (50 ppt) salinity for two weeks prior to gut microbiome analysis. **B)** Rarefaction curves demonstrate sufficient sequencing depth and reduced taxa richness in high-salinity fish relative to low-salinity controls. **C)** Alpha diversity analysis (Shannon Index) showing no significant difference in within-sample diversity between salinity treatments (p = 0.095). **D)** Bray-Curtis beta diversity analysis revealing significant differences in microbial community composition between low- and high-salinity fish (PERMANOVA, F = 7.09, p = 0.001). **E)** Phylum-level taxonomic composition of the gut microbiome, with high-salinity fish exhibiting increased dominance of Proteobacteria. **F)** Differential family-level abundance analysis identifying enrichment of Roseobacteraceae and Paracoccaceae in low-salinity fish and Comamonadaceae and Ahrensiaceae in high-salinity fish. **G)** Correlation of genus-level taxonomic assignments generated by Illumina and Oxford Nanopore sequencing, demonstrating strong agreement between platforms. **H)** Species-level relative abundance determined by Oxford Nanopore sequencing, showing enrichment of *Ahrensia kielensis* and *Vibrio scophthalmi* in high-salinity fish and a more evenly distributed microbial community in low-salinity fish.

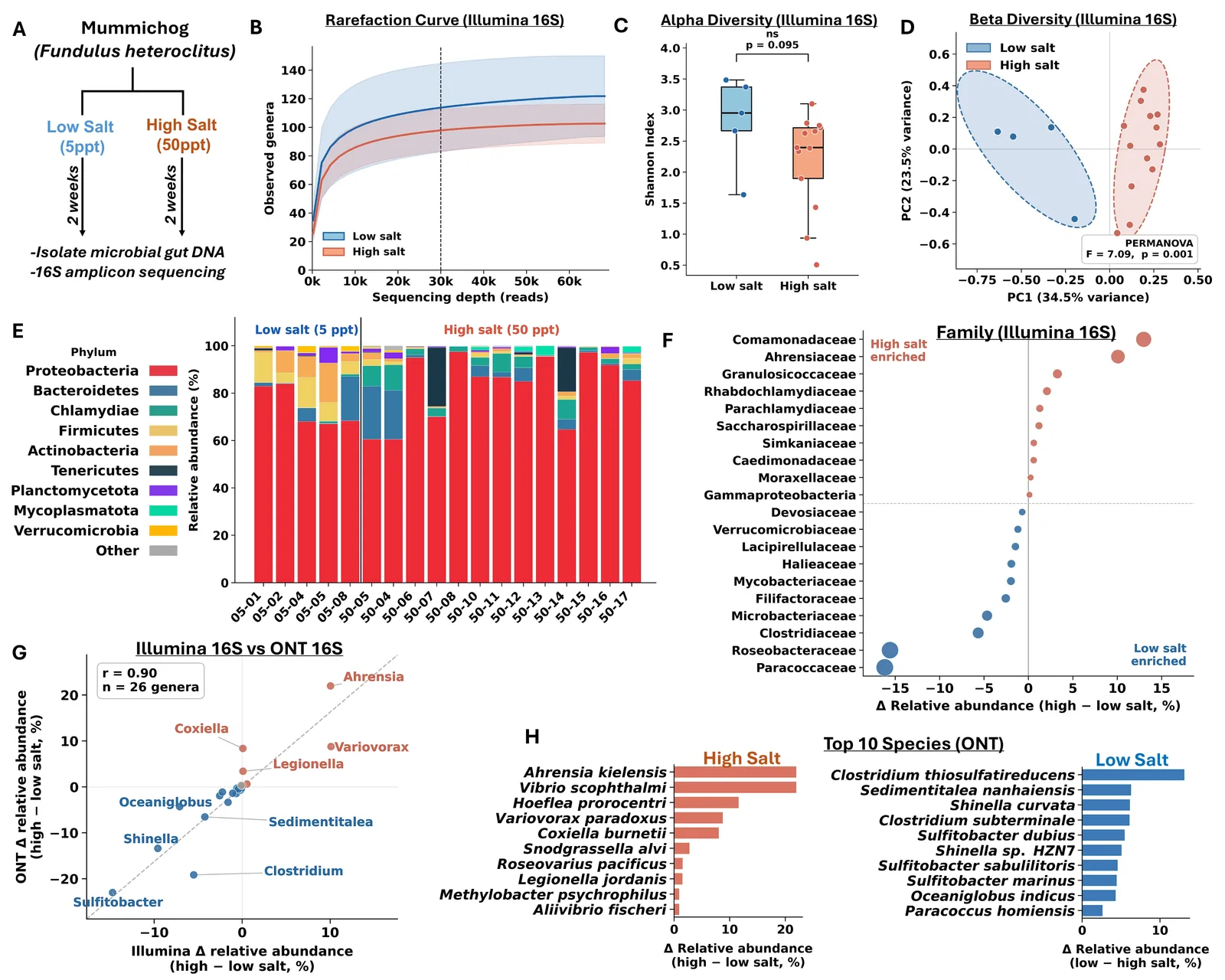



## Description

The gut microbiome is the microbial community, including viruses, archaea, and bacteria, inhabiting the digestive tract of animal hosts. These microbes form symbiotic relationships with their host, contributing to digestion, immune function, and metabolic processes, while benefiting from a stable nutrient-rich environment. Microbiome composition can be influenced by diet, age, environmental conditions, and host physiology, and these changes in microbial composition can in turn influence host phenotype (Dave et al. 2012, Evariste et al. 2019, Legrand et al. 2019, Macke et al. 2016). Because of its impact on numerous physiological processes, the gut microbiome has become an important area of research aimed at better understanding host health and responses to environmental change.

Mummichogs ( *Fundulus heteroclitus* ) are estuarine specialists that inhabit dynamic coastal ecosystems where freshwater and saltwater mix to form brackish environments. Salinity levels in estuaries can fluctuate due to tides, rainfall, and seasonal changes, requiring mummichogs to adapt quickly to maintain osmotic homeostasis. Their tolerance to wide salinity ranges makes mummichogs an intriguing model for studying physiological responses to osmotic stress (Telesh et al. 2010). Nevertheless, while these traits have made mummichogs an interesting animal model from an adaptive physiological perspective, there currently has been little effort in understanding how their physiological responses to these ranging salinity environments correspond with microbial changes within their gut.

Therefore to help bridge this disconnect between host physiology and the microbiome in mummichogs, we analyzed the effects of varying environmental salinities on the gut microbiome between mummichogs maintained under either high salt or low salt conditions and investigated how microbial taxonomic composition may contribute to host homeostasis under extreme salinity gradients. Two groups of mummichogs (age range 6 months-2 years; mixed sex) were examined where the control group was maintained in InstantOcean® at 5 ppt while the experimental group was maintained at a high salinity concentration of 50 ppt. After two weeks, the gut microbial DNA of both groups was isolated for both Illumina rRNA 16S amplicon sequencing and ONT full length 16S gene sequencing ( Figure 1A ). Of the 24 fish, five control and thirteen experimental fish met DNA quality and concentration thresholds and were retained for downstream analysis.

Based on the calculated rarefaction curves comparing sequencing depth and taxa identification ( Figure 1B ), the low-salinity control group showed consistently higher observed taxa richness across sequencing depths compared to the high-salinity group, with the two curves remaining separated throughout. Both rarefaction curves reached a plateau indicating sufficient sequencing depth to capture the majority of microbial diversity present. The separation between curves suggests that elevated salinity may exert a selective pressure on the gut microbiome, resulting in reduced species richness. Alpha diversity, which describes the variety and distribution of taxa within a sample, was assessed based on genus levels using a Shannon Index ( Figure 1C ). Although the low-salinity group exhibited slightly higher diversity values than the high-salinity group, the difference is not statistically significant (p=0.095). Therefore, salinity did not significantly affect the overall within-sample diversity of the gut microbiome.

Nevertheless, to further explore how salinity shapes community structure beyond overall richness, we examined beta diversity of genus levels, which measures the differences in microbial community composition between samples. A PCoA of Bray-Curtis dissimilarity revealed that the two treatment groups formed two distinct clusters, indicating that salinity drives differences in overall gut microbiome composition ( Figure 1D ). The clear separation between the two treatment groups suggests that salinity strongly influences the microbial composition of the gut microbiome. The Bray-Curtis PERMANOVA analysis with an F-value of 7.09 and a p = 0.001 confirmed the observed differences in microbial composition were statistically significant. These findings emphasize the influence of salinity on microbial composition in the gut microbiome of mummichogs. Together, the alpha and beta diversity analyses indicate that while overall microbial richness (alpha diversity) did not differ significantly between the salinity treatment groups, the specific taxonomic composition of the microbial communities (beta diversity) differed significantly.

We then examined the taxonomic composition of the gut microbial communities in both treatment groups. From these results, we observed a shift toward Proteobacteria-dominated communities in high-salinity fish, whereas low-salinity fish exhibited a more balanced phylum distribution suggesting that salinity selects for more specialized microbial taxa ( Figure 1E ). To further study microbiome composition shifts associated with salinity, we analyzed differential relative abundance at the family level ( Figure 1F ). Here, families such as Roseobacteraceae were significantly enriched in gut microbiomes of low-salinity fish, whereas Comamonadaceae were significantly enriched in high salinity fish. Roseobacteraceae are known to contribute to metabolism of organic matter and may play roles in protecting against harmful pathogens. In contrast, Comamonadaceae are associated with metabolic versatility and responses to environmental stress (Buchan et al. 2022, Luo et al. 2014, Willems et al. 2014).

The enrichment of these families in high-salinity fish suggests that elevated salinity may select for microbes better adapted to halotolerant environments, potentially contributing to host adaptation to salinity stress. To further determine the taxonomic differences of the gut microbial communities between the low- and high-salinity fish, we next compared the relative abundance of bacterial genera identified by Oxford Nanopore long-read sequencing and Illumina short-read sequencing ( Figure 1G ). A strong correlation between Oxford Nanopore and Illumina sequencing results shows consistency between the two platforms and supports the accurate identification of bacterial genera within the mummichog gut microbiome. Because Oxford Nanopore sequencing captures the full-length 16S rRNA gene (1.5kb), species-level resolution was achieved. Species level analysis of relative abundance showed low-salinity fish maintained a more evenly distributed microbial community, whereas high-salinity fish were characterized by an abundance of a few species, particularly *Ahrensia kielensis* and *Vibrio scophthalmi* . These species are marine-associated bacteria that possess specialized mechanisms for survival in high-salinity environments. One key adaptation is the presence of Na⁺-NQR, an enzyme that uses electron transport energy to actively export sodium ions out of the cell, supporting ion balance and cellular energy processes under high salinity conditions. The enrichment of bacteria containing the Na⁺-NQR enzyme in the fish gut microbiomes suggests that salinity selects microbial taxa that are capable of tolerating osmotic stress (Genz & Grosell 2011, Gregory et al. 2021, Liu et al. 2016, Barquera et al. 2014).

This study demonstrates that environmental salinity significantly influences the composition of the mummichog gut microbiome. While alpha diversity did not differ significantly between treatment groups, beta diversity analyses revealed distinct microbial communities in low- and high-salinity fish. High salinity was associated with an enrichment of specific bacterial taxa, including *Ahrensia kielensis* and *Vibrio scophthalmi* , showing a transition toward bacteria adapted to elevated salt conditions. These findings highlight the dynamic relationship between environmental salinity and gut microbiome composition and support the use of mummichogs as a valuable model for studying microbiome interactions in response to environmental stress. Similar conclusions have been observed in previous studies of mummichogs exposed to other environmental stressors, including PCB-126 exposure, which also resulted in significant shifts in gut microbiome composition (Ma et al 2025). Together, these studies emphasize the impact of the environment on the mummichog gut microbiome. Future studies could investigate whether the Na⁺-NQR-containing bacteria enriched under high salinity functionally contribute to host osmoregulation, further clarifying the role of the microbiome in mummichog adaptation to extreme salinity gradients. Additionally, future studies examining the temporal dynamics of gut microbiome recovery following salinity transitions could further clarify the stability and resilience of these microbial communities under fluctuating estuarine conditions.

## Methods

*Mummichog care and experimentation*

Mummichogs (Fundulus heteroclitus, n=24) of similar age ranging from 6 months to 2 years of age were obtained from Atlantic Aquaculture (https://atlanticaquaculture.co/) and maintained on a diet of Ziegler Brothers Adult Zebrafish Food. All animal procedures were approved by the Institutional Animal Care and Use Committee (IACUC) of Presbyterian College (Protocol # 03-22-2025) and were conducted in accordance with institutional guidelines for the care and use of laboratory animals. Fish were randomly divided into two tanks containing approximately 12 fish in each and acclimated to either low salinity (5 ppt InstantOcean®, the control group) or high salinity (50 ppt InstantOcean®, the experimental group). InstantOcean was used as the artificial sea salt to establish salinity conditions while minimizing potential environmental contamination from natural seawater. After two weeks of treatment, the fish were sacrificed and dissected. An incision was made from the ventral surface to expose the digestive tract, and gut contents were collected into a 150 µL lysis buffer slurry for DNA extraction. Genomic DNA was extracted using the ZymoBIOMICS DNA Miniprep Kit according to the manufacturers' protocols. DNA concentrations were quantified using the Quant-iT™ PicoGreen™ dsDNA Assay according to the manufacturer's instructions. Samples with DNA concentrations greater than 2.1 ng/µL were selected for downstream sequencing analyses.

*Gut contents analysis*

DNA samples of five control fish and thirteen experimental fish meeting quality and concentration requirements were submitted to MR DNA (Shallowater, TX, USA) for 16S rRNA amplicon sequencing targeting the V4 hypervariable region to characterize bacterial community composition. A separate set of DNA samples was submitted to Plasmidsaurus (San Francisco, CA, USA) for full-length Oxford Nanopore 16S rRNA gene sequencing to enable species-level taxonomic identification.

*Taxonomic profiling via Illumina sequencing*

Sequenced DNA was processed using QIIME2 version 2026.4 (Boylen et al. 2019) to generate taxonomy-resolved read count tables from kingdom to genus level. Resulting count tables were further analyzed in Python (pandas, NumPy, SciPy, statsmodels, matplotlib) to generate the statistics and visualizations described below. Rarefaction curves were generated for each sample to assess sequencing depth and confirm that microbial diversity had been adequately sampled; based on these curves, samples were rarefied to a common depth of 30,000 reads prior to downstream diversity analyses. Alpha diversity was assessed at the genus level using the Shannon diversity index, and differences between salinity treatments were evaluated using a two-sided Mann-Whitney U test. Beta diversity was assessed using Bray-Curtis dissimilarity calculated on rarefied genus-level relative abundance data, and community structure was visualized using principal coordinates analysis (PCoA). Differences in community composition between salinity treatments were tested using permutational multivariate analysis of variance (PERMANOVA) with 999 permutations. Relative abundance bar graphs were generated to visualize taxonomic composition at the phylum level for each sample. The first five samples represent the control (low salinity) group (05-01, 05-02, 05-04, 05-05, 05-08), and the final thirteen represent the experimental (high salinity) group (50-04, 50-05, 50-06, 50-07, 50-08, 50-10, 50-11, 50-12, 50-13, 50-14, 50-15, 50-16, 50-17).

*Differential abundance analysis*

Differential abundance between salinity treatments was assessed at the genus and family levels using rarefied count data. For each taxon, relative abundance was compared between treatment groups using a two-sided Mann-Whitney U test, and p-values were adjusted for multiple comparisons using the Benjamini-Hochberg false discovery rate (FDR) procedure. Taxa were retained for testing if they were present in at least two samples within either treatment group and had a mean relative abundance of at least 5 reads (post-rarefaction). Taxa with an adjusted p-value (FDR) < 0.05 were considered significantly differentially abundant. Effect size was expressed as the delta (difference) in mean relative abundance between high- and low-salinity groups.

*Species-level taxonomic profiling via Oxford Nanopore sequencing*

To obtain species-level taxonomic resolution beyond that achievable with short-read 16S amplicon sequencing, pooled DNA samples from high-salt and low-salt treatment groups were submitted to Plasmidsaurus for long-read 16S rRNA gene sequencing on the Oxford Nanopore platform, returning basecalled FASTQ files. Reads were separated into high-salt and low-salt datasets prior to taxonomic classification. Taxonomic classification was performed independently for each treatment group using EMU v3.6.1 (Curry et al. 2022), which estimates relative abundance at the species level by aligning long reads to a reference database (the EMU default database derived from NCBI 16S rRNA sequences) using minimap2 and resolving multi-mapping reads via an expectation-maximization algorithm.
